# Mobile Dental Clinics: Bringing Smiles on Wheels

**DOI:** 10.7759/cureus.83873

**Published:** 2025-05-11

**Authors:** Minal Kshirsagar, Arun Dodamani, Sandeep Pimpale, Sanpreet S Sachdev, Dipooja Patil, Mahesh Ghadage, Uttam Shetty, Hritika Khobrekar

**Affiliations:** 1 Public Health Dentistry, Bharati Vidyapeeth (Deemed to be University) Dental College and Hospital, Mumbai, IND; 2 Public Health Dentistry, Jawahar Medical Foundation's ACPM Dental College and Hospital, Dhule, IND; 3 Periodontics, Nair Hospital Dental College, Mumbai, IND; 4 Oral Pathology and Microbiology, Bharati Vidyapeeth (Deemed to be University) Dental College and Hospital, Mumbai, IND; 5 Conservative Dentistry and Endodontics, Bharati Vidyapeeth (Deemed to be University) Dental College and Hospital, Mumbai, IND; 6 Prosthodontics and Crown and Bridge, Bharati Vidyapeeth (Deemed to be University) Dental College and Hospital, Mumbai, IND; 7 Prosthodontics, Bharati Vidyapeeth (Deemed to be University) Dental College and Hospital, Mumbai, IND

**Keywords:** community outreach, mobile dental clinics, preventative dental treatments, public-private collaboration, rural oral healthcare

## Abstract

The present narrative review explores the role and impact of mobile dental clinics in improving access to oral healthcare for underserved populations in India. The primary objective is to assess how mobile dental services bridge the gap in dental care delivery between urban and rural regions by addressing geographical, financial, and informational barriers. Mobile dental units (MDUs), equipped with diagnostic and treatment facilities, extend comprehensive care, including oral examinations, hygiene education, preventive treatments, and basic restorative procedures to remote and marginalized communities. These clinics function as community outreach platforms, often operated by dental colleges and public health programs, to promote oral health awareness and provide cost-effective care. Mobile dental units demonstrate higher reach and engagement than stationary clinics, contributing significantly to improved oral health outcomes among target populations. Governmental and institutional support through policy initiatives and public-private partnerships further enhances the scalability and sustainability of such services. This review underscores the growing potential of mobile dentistry in India and calls for continued innovation, strategic planning, and technological integration to ensure equitable and inclusive oral healthcare delivery.

## Introduction and background

India faces major public health challenges due to insufficient dental care access, which affects most of its population. A majority of Indian citizens reside in rural communities, whereas most dental facilities and professionals are concentrated in urban centers. The current dentist-to-population ratio in India stands at 1:10,000, whereas the World Health Organization recommends 1:7,500 clinicians per population, and approximately 80% of Indian dentists practice within urban areas [1,2]. A recent report by the Indian Dental Association further highlights that more than 75% of dental clinics are located in urban or semi-urban areas, serving only about 30% of the population, while the remaining 70%, predominantly in rural zones, have access to less than 25% of the available dental infrastructure [2].

This urban-rural imbalance, combined with high levels of poverty and low health literacy in rural areas, leads to a large unmet need for oral healthcare. Studies have shown a rising burden of dental diseases in these underserved populations, including high prevalence of dental caries and periodontal disease, and India has been dubbed the “oral cancer capital” of the world due to widespread tobacco use [3]. Yet, people in remote or low-income groups often go without treatment; for example, one rural outreach program found over 93% of patients had never received any prior dental care [4].

Barriers to dental care in India include geographic isolation, lack of transportation, financial constraints, and social factors. Rural residents may need to travel long distances to reach a dentist, which is difficult due to inadequate public transport and challenging terrain [5]. The cost of dental treatment in private clinics is prohibitive for lower socio-economic groups, leading many to delay or avoid care until problems become severe. Government dental facilities are limited and serve only a small fraction of the patients, while the majority must rely on private practitioners [6]. Consequently, untreated dental issues can progress to advanced disease, causing pain, tooth loss, and even systemic health complications. Innovative strategies are needed to deliver oral healthcare to those who cannot access conventional clinics [7].

Mobile dental clinics, also referred to as 'mobile dental vans' and 'mobile dental units' (MDUs), act as an effective remedy for delivering oral healthcare services in underserved locations. These dental clinics on wheels enable on-site dental service delivery using specially equipped trailers, trucks, and buses outfitted with dental chairs, diagnostic tools, and materials for patient care [8]. This mobility enables dental providers to reach patients in remote villages, urban slums, educational institutions, and community sites. Mobile dental services help make healthcare more accessible and cost-effective by overcoming physical distance and transport-related barriers [9]. These outreach-based interventions are being increasingly implemented not only in India but globally, and are recognized as a promising solution to healthcare inequity.

Health authorities and dental institutions endorse the mobile dental service concept in India. Community dentistry programs and dental camps are supported by mobile clinic vans operated by dental colleges, which serve distant populations while also providing training platforms for undergraduate students [10]. The Dental Council of India mandates that dental colleges maintain outreach MDUs as part of their public health mandate. In addition, various government and non-government organizations utilize MDUs to deliver free oral healthcare across rural and urban poor regions. These efforts align with India’s broader Universal Health Coverage objectives, which aim to provide essential oral healthcare to marginalized and geographically isolated populations.

The present narrative review aims to critically explore the role of mobile dental clinics in bridging the oral healthcare gap in India. The primary objectives are to (1) evaluate the operational effectiveness of MDUs in reaching underserved populations, (2) analyze their cost-efficiency and service coverage compared to conventional clinics, (3) examine the integration of mobile dental services within existing public health and policy frameworks, and (4) identify implementation challenges and opportunities for technological and strategic enhancement. By synthesizing available literature and government program data, the review aims to provide actionable insights that can guide policymakers, public health planners, and dental institutions toward scaling up mobile dental services in an evidence-based and equitable manner.

## Review

Methodology

The search for relevant literature concerning the use of mobile dental vans in India was performed using the keywords 'Mobile Dental Van' OR 'Mobile Dental Clinic' AND 'India' across various databases, including PubMed, SCOPUS, ScienceDirect, EMBASE, EbscoHost, and Google Scholar. Articles published after the year 2000 (the past 25 years) with full text available in the English language were selected. Cross-sectional studies and review articles concerning the mobile dental clinics/vans in India were included, while letters to the editor, conference proceedings, and technical notes were excluded. The medical subject headings (MeSH) term search included keywords "Dental Clinics"[MeSH Terms], AND "Dental Care"[MeSH Terms], AND "Public Health"[MeSH Terms], AND "Delivery of Health Care"[MeSH Terms], which yielded a total of n=142 relevant articles.

The identified titles were screened by the authors MK and SSS, following which full texts of the eligible studies were downloaded. The data reported in these articles were segregated into various domains listed as subheadings in the present review. Relevant information was entered under the corresponding subheadings by all the authors after a thorough reading of the articles. Final data analysis and synthesis were performed by authors AD, SP, DP, and MG.

Importance of mobile dental clinics in India

**Figure 1:**
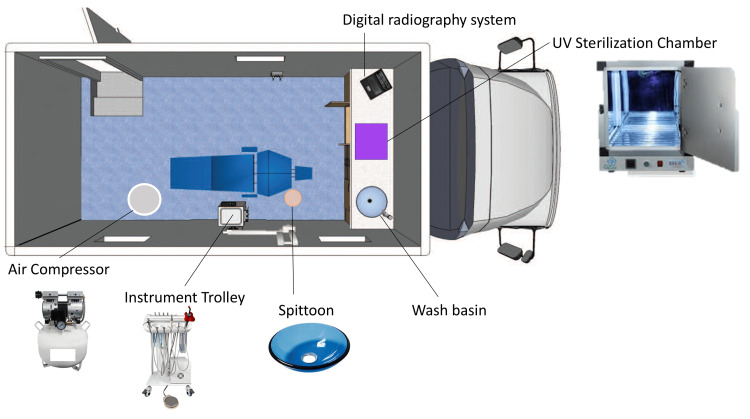
Components of a workstation in a mobile dental van Image courtesy: Author Sanpreet S. Sachdev

Mobile dental clinics play a crucial role in improving access to oral healthcare for underserved populations in India. They are particularly important in overcoming geographic and socio-economic barriers that prevent people from obtaining dental care [1]. By bringing services directly to remote and underprivileged communities, mobile clinics effectively “reach the unreached.” These units have been shown to increase utilization of dental services among groups that typically have limited access, such as rural villagers, urban slum dwellers, children, the elderly, and other vulnerable populations​ [11]. Unlike stationary clinics that require patients to travel, mobile clinics travel to convenient locations in the community, making it far more likely that people will seek and receive care ​[5]. This flexibility allows MDUs to address geographic and even cultural barriers; for example, they can operate on specific days in a village, align with local events, and build trust within the community. The components of a mobile dental van are depicted in Figure 1.

One of the key advantages of MDUs is their ability to reduce the inequality in oral healthcare between urban and rural areas. John et al., in their review, asserted “MDUs can be a catalyst in eliminating rural-urban inequality in terms of dental care” [12]. Rural communities, low-income groups, migrant laborers, and homeless populations all benefit from the outreach provided by mobile clinics​ [13]. These services ensure that people who cannot afford private dental care or travel to distant clinics are not completely left without care. For children in isolated areas, school-based mobile dental programs provide a rare opportunity for dental check-ups and treatment, thereby improving their oral health outcomes​ [13]. Similarly, mobile units visiting nursing homes or community centers can deliver care to the elderly or disabled who would otherwise face great difficulty in visiting a dentist.

Mobile dental clinics serve as valuable supplementary services to the present oral healthcare facilities. These facilities jointly operate with dental colleges and district hospitals, and NGOs to provide extended community outreach for institutions [9]. Research findings demonstrate that MDUs bring better accessibility combined with affordability, thus reaching larger numbers of individuals than conventional fixed-clinic settings [9]. Furthermore, government health departments can deliver cost-effective care through mobile van services to populations who need them most [13]. Mobile dental clinics operate as mini-dental institutions for locations without permanent facilities where basic preventive education and treatment are accessible on-site. Mobile clinics both relieve dental health discomfort and teach dental awareness to patients so they understand when to seek further sophisticated dental care.

In addition to dental problem treatment, mobile clinics support community health through diverse means. The dental teams on mobile units use the initial contact to deliver valuable oral health education about proper dental practices combined with nutrition guidance against dental decay and tobacco use prevention strategies to rural communities [9]. Several underprivileged communities lack basic oral health knowledge, which makes mobile dental camps essential for raising awareness about good dental practices among people unfamiliar with dentists. The educational component proves essential because India lacks scientific dental understanding, which otherwise could lead to dental problem dismissal. Mobile dental clinics fulfil a dual purpose by offering medical intervention and educational disabilities treatment to patients [10].

The MDUs function to expand the availability of dental professionals across different locations. Mobile dental clinics establish channels through which dental experts can choose to deliver temporary services in rural locations. For instance, dental colleges send interns and postgraduates to rural postings using mobile vans, which not only provide care to villages but also give young dentists exposure to community dentistry. Some mobile clinic programs allow urban-based dentists to participate periodically without requiring permanent relocation, helping address the shortage of providers in rural regions [4]. In return, those dentists gain experience and satisfaction from community service, and the community gains access to professional care. This model of outreach thus helps utilize the growing number of dental graduates in India for public health benefits [14].

Services provided

Mobile dental clinics in India are equipped to deliver a range of essential dental services, focusing on preventive and basic curative care that addresses the most common needs. Typically, these mobile units are designed as self-contained operatories with one or more dental chairs, instrument setups, and materials to perform routine dental procedures​ [8]. Although compact, they often carry all necessary equipment such as compressors, suction units, autoclaves for sterilization, and portable X-ray units if space permits​ [15]. This enables mobile clinics to function almost like a small dental clinic. The services provided by mobile dental clinics are listed below.

Oral Examination and Diagnosis

Mobile camps begin with dental check-ups for patients. Dentists take patient histories, perform oral examinations, and diagnose common issues such as dental caries, gum disease, oral infections, and suspicious lesions. Screening for oral cancer is also commonly done, especially in areas with high tobacco use. Basic diagnostic tools and sometimes portable radiographic equipment allow identification of problems on-site [1].

Preventive Services

Preventive dental care is a cornerstone of mobile clinic programs. Services often include dental cleanings (scaling and polishing) to remove plaque and tartar, topical fluoride applications to strengthen enamel, and placement of pit and fissure sealants on children’s teeth to prevent cavities. Oral hygiene instructions are provided to individuals and groups, teaching proper brushing techniques and flossing. Health education is a continuous activity - the mobile clinic staff use visual aids, models, and demonstrations to promote good oral health practices in the community​ [1]. In some programs, mobile units also coordinate with general health services to provide immunizations (e.g., for children) and nutritional counseling as part of an integrated preventive approach.

Restorative Treatments

Mobile dental units can perform basic restorative procedures to treat dental caries and alleviate pain. This includes placing fillings (restorations) using materials like dental amalgam or composite resin to restore decayed teeth​ [4]. The clinics are equipped with dental handpieces (drills) and restorative instruments to remove decay and fill cavities. They maintain supplies of filling materials, liners, and basic dental lab tools necessary for such treatments. By providing fillings on-site, mobile clinics prevent small cavities from progressing and save teeth that might otherwise be lost due to a lack of timely care.

Dental Extractions

Tooth extractions are one of the most frequently provided services, given that many patients present with badly decayed or infected teeth that are beyond simple restoration. Mobile clinic dentists perform simple extractions of loose or non-salvageable teeth under local anesthesia​ [4]. They carry anesthetic supplies and instruments such as forceps and elevators to safely remove teeth. This service is critical for relieving pain and preventing the spread of dental infections, especially in communities where advanced dental treatments are not accessible. However, mobile setups generally restrict extractions to simple cases; impacted wisdom teeth or surgical extractions requiring sophisticated equipment are referred to a hospital.

Basic Endodontic and Minor Surgical Procedures

Depending on the resources of the van, some mobile clinics also conduct basic endodontic procedures such as pulpotomies or even root canal treatment for anterior teeth, and minor oral surgeries. For example, one innovative mobile clinic model in India (a trailer unit) was able to provide minor oral surgery procedures in the field. In general, procedures like draining abscesses, treating gum infections, and performing minor gum surgeries can be done. Anything requiring a fully sterile operating environment or advanced surgical instruments is deferred due to the limitations of the mobile setting​ [16].

Oral Health Education and Promotion

A significant portion of the mobile clinic’s mission is devoted to education and awareness. Many MDUs have an attached area or provision for group education; for instance, a waiting area that doubles as an oral health education center with posters, audio-visual equipment, and models. While patients wait for treatment or after receiving care, dental staff engage them in talks about preventive oral care, demonstrate brushing techniques, and discuss topics of diet and tobacco cessation. Some programs also involve community health workers or teachers (in school camps) to reinforce oral health messages [1]. This educational function helps create a lasting impact beyond the immediate treatments given.

**Figure 2:**
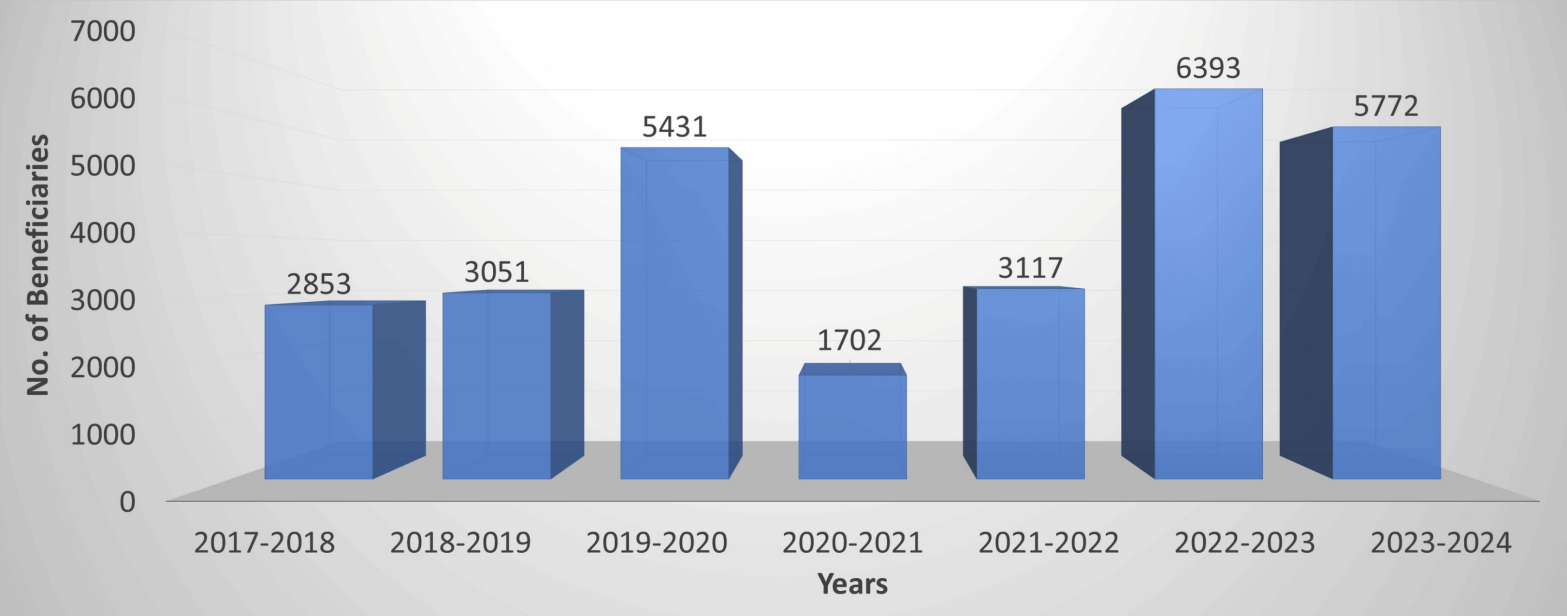
Beneficiaries of our institute's MDU under the National Service Scheme MDU: Mobile dental unit Graph created by Author Sanpreet S. Sachdev

Mobile dental clinics, thus, provide comprehensive primary dental care services [17]. An illustrative example comes from a three-year mobile dental program in rural India, which delivered an array of services at no cost to patients: dental examinations, cleanings, fluoride treatments, sealants, restorations, extractions, and even minor oral surgeries were all provided on-site to thousands of villagers. Notably, that program treated 6,326 patients over three years and uncovered that the vast majority had never received dental care before, underscoring the value of such outreach​ [4]. Likewise, about 28,319 beneficiaries could avail of dental treatment due to the community outreach programme of our institute under the National Service Scheme (Figure 2).

In addition to direct patient care, mobile clinics often serve in community programs such as school dental health camps and rural health fairs. For instance, some mobile units visit schools weekly to conduct dental check-ups for children and apply fluoride or sealants as needed. By operating in schools, they capture children who might otherwise never see a dentist and provide referral slips for those needing further treatment. Mobile clinics also partner with local healthcare providers to integrate oral health with general health check-ups, so that dental care becomes a part of primary healthcare in those communities.

It is important to note that while mobile dental clinics cover a broad range of basic services, they do have limits. Procedures that require specialized equipment (like full denture fabrication, complex root canals, orthodontics, or advanced surgeries) are generally not done on the van​ [16]. Patients needing such care are usually referred to the nearest dental hospital or clinic. Nevertheless, by handling the bulk of common dental problems and preventive needs, mobile clinics substantially reduce the burden of untreated disease. They address the immediate dental needs of the community and lay the groundwork for a preventive oral health culture. All these services are delivered with minimal infrastructure on-site, showcasing the efficiency and reach of mobile dentistry in the Indian healthcare context.

Cost-effectiveness

One of the compelling advantages of mobile dental clinics is their cost-effectiveness in delivering oral healthcare to dispersed populations. From the perspective of healthcare providers and policy planners, mobile units can often provide services at a lower cost per patient compared to establishing and running permanent clinics in every remote location​ [13,18]. Several factors contribute to the cost-effectiveness of mobile dental clinics in India.

First, mobile clinics allow optimal utilization of resources by moving the service to areas of need. Instead of building multiple stationary clinics that might remain underutilized in sparsely populated areas, a single MDU can serve many villages on a rotating schedule. This flexibility means the expensive equipment (dental chair, instruments, etc.) and the dental team’s time are maximized, treating patients in different localities on different days. Studies have noted that MDUs, when adequately utilized, can treat more people than equivalent fixed clinics with the same resources​[4,18]. In essence, one van can do the work of several small clinics by covering a larger catchment area, which is economically efficient.

Second, the initial investment and overhead costs for an MDU can be lower than setting up a new dental facility [19]. Setting up a brick-and-mortar dental clinic involves costs of land or building rental, construction, utilities, and permanent staffing. In contrast, a mobile van, while having a significant upfront cost for the vehicle and equipment, can be seen as a one-time capital investment that eliminates the need for multiple physical buildings. Hospitals and organizations have favoured mobile clinics because of their high mobility and relatively lower cost compared to establishing new clinics in each location​ [14,19]. Maintenance and operation costs exist (fuel, vehicle upkeep, salaries), but these can be budgeted in a centralized manner. One analysis suggested that mobile dental clinics were found to be more cost-effective in providing child dental services than traditional public or private clinics, when comparing the cost per child treated​ [9]. Mobile clinics also often leverage existing infrastructure (like parking at a local school or community center) rather than bearing facility costs.

Third, mobile dental programs frequently operate on a prevention-focused model, which can lead to long-term cost savings in oral healthcare. By reaching people early and providing preventive care (fluoride, sealants, education) and simple treatments, mobile clinics help avoid more serious dental problems that would be costlier to treat later in hospitals. Preventing a cavity or extracting a decayed tooth before it causes an abscess is not only better for the patient but also averts the higher expenses of emergency care or complex treatments. Although it is hard to quantify these averted costs directly, public health experts recognize that bringing basic care to underserved areas can reduce the overall burden and expense of advanced disease management in the healthcare system​ [9].

From the patient’s perspective, mobile clinics significantly cut down the indirect costs of seeking care, which adds to their cost-effectiveness in a societal sense. When dental services come to the village, patients save on travel expenses, which in rural India can be substantial (bus fares, long journeys), and on lost wages or time since they do not need to take as much time off work. This makes the utilization of services higher, as noted earlier, and ensures that money is not a barrier for the poorest segments (especially when services are provided free or at nominal cost during mobile camps)​. In government-run mobile dental programs or NGO initiatives, care is often provided at no charge to the community, meaning the cost is absorbed by the program, and it can often operate on a modest budget. Many mobile units in India are funded through public health budgets, university grants, or corporate social responsibility (CSR) funds, making them essentially a low-cost or free service for end-users [15]. By reducing out-of-pocket expenses, these programs make dental care more equitable.

Evidence of cost-effectiveness can be seen in practice. The Indian Army and some state governments have operated mobile dental buses that report high volumes of patients at relatively low running costs, although detailed cost analyses are not always published. In one review of various programs, it was reported that mobile dental clinics tend to offer services “at lower or no cost to the user” and thereby remove the financial barrier that is a major issue in conventional dental practice​ [19]. Another study concluded that when comparing the costs of providing school dental care via a mobile clinic versus sending children to private clinics, the mobile clinic model was financially favourable in terms of cost per child treated [20]​.

It should be noted that the cost-effectiveness of mobile clinics can depend on maintaining a high level of utilization and good logistical planning. The upfront cost of a well-equipped dental van is high, and if the vehicle is not used frequently or sits idle, the cost per patient will increase [21]. Additionally, fuel and maintenance costs can rise if the distances are large. To maximize efficiency, scheduling and routing of mobile dental services are often done strategically; by clustering villages and schools, the MDU can serve many people in one trip, and ensure that each visit attracts a good number of patients through prior community mobilization. When properly organized, the benefits of scale and outreach outweigh the expenses.

In summary, mobile dental clinics offer a cost-effective approach to extend oral healthcare in India. They reduce the need for multiple static clinics, lower infrastructure and operational costs, and deliver prevention and early treatment that can save costs in the long run. By leveraging existing health systems and focusing on high-need areas, they provide “affordable dental care to the underserved”, as evidenced by government and institutional experiences​ [14]. As India looks to improve healthcare access, the mobile clinic model in dentistry stands out as a financially sensible strategy to reach remote populations without a prohibitive price tag.

Challenges and limitations in the Indian context

While mobile dental clinics are invaluable in expanding access to care, they face several challenges and limitations, especially in the context of India’s diverse and resource-constrained settings. Understanding these limitations is important for optimizing mobile dental programs and making them sustainable. The key challenges are featured below.

Limited Scope of Treatment

By design, MDUs focus on basic dental services and have constraints in offering advanced or specialized treatments. The confined space and portable equipment in a van are sufficient for examinations, fillings, cleanings, and simple extractions, but not for complex procedures [15]. Treatments that require a completely sterile environment or specialized machinery (such as major oral surgeries, complex root canals, orthodontics, or treatment of oral cancer lesions) cannot be performed on the MDU. As Grover et al. explained, expecting procedures like surgical excision of a tumor or multi-specialist treatments in a small mobile setup is unrealistic​ [1]. Such cases still need referral to equipped hospitals. This limitation means mobile clinics must operate as part of a referral network. They can identify and start managing a problem, but patients with advanced needs may have to travel to a tertiary center, which can be a barrier if follow-up is not ensured [15].

Infrastructure and Maintenance Issues

Keeping a mobile dental clinic operational requires maintenance and technical support. The vehicles endure long travel on often poor rural roads, leading to wear and tear. Breakdowns of the van or failure of onboard equipment (compressors, generators, dental chairs) can interrupt services. An experienced maintenance team is essential to repair mechanical or electrical issues promptly​ [22]. However, not all programs have dedicated technicians as staff, and repairs can be costly or slow in remote areas. Additionally, the van relies on certain utilities: power (usually through a generator) and water supply for dental procedures [22]. Managing fuel for the generator, safe water storage, and waste disposal are logistical challenges that require careful planning. These operational complexities can strain the budget and management capacity of mobile clinic programs.

High Operating Costs and Funding Constraints

Although mobile clinics are cost-effective in broad terms, they do involve significant operating costs. The initial purchase of a dental van with equipment is expensive, often only affordable to institutions, governments, or large organizations. Individual private dentists find it financially unfeasible to buy and run an MDU on their own. Beyond initial costs, recurring expenses include fuel (which can be substantial given the long distances traveled), vehicle maintenance, consumable dental materials, and salaries for the dental team and driver/coordinator [23]. If the program relies on grants or government funds, there may be budget limitations that restrict how often and how far the MDU can travel. Ensuring a continuous supply of materials (fillings, anesthetics, disposables) is another cost factor. Without steady funding or revenue, some mobile clinics struggle to maintain regular operations [24]. For example, an MDU might only go to a village a few times a year due to limited funds for petrol or staff, reducing its impact.

Human Resource Challenges

Mobile dental clinics typically require a dedicated team willing to work in field conditions. Dentists and staff may have to travel long hours, work in modest environments (sometimes without the comfort of a full clinic setup), and adapt to varying field conditions (heat, dust, lack of amenities). Attracting skilled dental professionals to work in rural mobile units can be difficult, especially given higher-paying or more comfortable opportunities in urban practices [25]. Many programs mitigate this by using interns or junior dentists as part of training, but high turnover can occur as they move on in their careers. Language and cultural barriers can also arise if the dental team is not local to the community being served. Additionally, the safety and security of the team and equipment while traveling to remote areas can be a concern, especially for night travel or politically unstable regions.

Community Awareness and Utilization

Simply deploying a mobile clinic does not guarantee that people will use the service. In some areas, initial utilization of mobile dental vans has been reported as low due to a lack of awareness or trust. Communities not familiar with dental care might be hesitant to seek treatment from a van that arrives occasionally. This indicates that mobilizing the local population through village leaders, health workers, announcements, and education is crucial for the clinic’s success. Overcoming cultural misconceptions (for example, fears about dental treatment) requires time and consistent presence. If a mobile van visits too infrequently or without local promotion, many target beneficiaries may remain unreached due to low turnout.

Continuity of Care and Follow-Up

Mobile dental programs often operate in a camp or periodic visit model, which can pose issues for continuity of care. Patients who receive a procedure might need a follow-up check or further treatment after some weeks, but the van may return only after a long interval. Ensuring that patients complete multi-step treatments (such as completing all sessions of a root canal or getting a prosthesis after an extraction) is challenging. Some programs address this by scheduling return visits and maintaining records to recall patients, but the logistics can be complex when dealing with moving operations and communities with no fixed addresses. Also, if a patient is referred to a hospital for advanced treatment, there is often no mechanism to ensure they go and receive treatment; many might default due to the same barriers initially present. This limitation means that while mobile clinics provide a great first line of care, integration with the wider health system is needed for comprehensive care.

The Indian government maintains no central authority to track mobile dental clinic distribution as well as their achievements through a database or regulatory system. The Dental Council of India, along with the Ministry of Health, does not establish routine procedures for data collection regarding MDU operations, target populations, and service delivery protocols. The system enables quality control primarily to rest with the responsible institutions that operate the clinics. It becomes challenging to maintain infection control and sterilization, together with proper record-keeping standards, for mobile clinics because they work beyond standard clinic environments. Some regions face obstacles related to legal requirements as well as administrative rules that require permission to park vans at sites and distribute medication. Organizations must develop standards for managing the expansion of mobile clinics, including equipment maintenance and biomedical waste disposal protocols, especially as it is in the developmental phase in India.

The mobile dental clinic system in India faces both technical problems regarding funding and maintenance as well as long-term technical problems with integration and community participation. Mobile dental clinics retain their worth despite the need to improve specific areas. The execution of mobile dental clinic solutions requires funding partnerships between public and private organizations, together with planned routine service schedules and involved community outreach programs supported by updated advanced medical technology in clinic setups. Proper backing through partnerships with local NGOs and government funding support allows many mobile clinic obstacles to become manageable [26]. The process of identifying constraints enables mobile dental services to achieve better sustainability alongside enhanced effectiveness in the long term.

Government initiatives and policy support

Many Indian government bodies, together with policy-making organizations, have bolstered their support for mobile dental clinics that reach vulnerable communities. The National Mobile Dentistry Program does not stand alone. Similarly, the mobile oral health services gain promotion through multiple government health schemes, along with educational requirements for dentists and NGO partnerships [26]. Various initiatives demonstrate how government institutions support mobile dental clinics through policy measures.

The Dental Council of India (DCI) requires dental colleges nationwide to maintain MDUs for the mandatory educational and community service facilities requirement. As a result, there are more than 300 educational institutions that can operate at least one mobile dental service clinic. Educational establishments use these vans to operate student and faculty groups during rural dental camps, along with school health programs and outreach activities as part of their Public Health Dentistry training. According to the DCI's requirements, public dental practitioners can operate in every region of India while dental graduates receive proper training in community dental care. The requirement generates more mobile dental clinics that cater to public health needs as colleges link up with local authorities to conduct rural dental camps.

The Ministry of Health & Family Welfare launched the National Oral Health Programme (NOHP) in the mid-2010s to enhance oral healthcare delivery as part of primary healthcare [27]. While the NOHP focuses on the integration of oral health into existing healthcare and improving dental facilities at district and community health centers, it also emphasizes outreach activities. Operational guidelines of NOHP encourage the use of dental outreach camps and mobile clinics to provide preventive and basic curative services in rural and tribal areas where dental infrastructure is lacking. For instance, the NOHP highlights that in the absence of established clinics, MDUs are a viable option to deliver services at the grassroots level. Under the NOHP, financial support is provided to states and union territories for the establishment of dental care units, including MDUs, at various levels of the healthcare system. This support encompasses funding for equipment, consumables, training, information, education, and communication activities, as well as the recruitment of manpower. The implementation of these initiatives varies by state and is often carried out in collaboration with dental colleges or district health societies. The NOHP recognizes MDUs as a viable solution to deliver oral health services at the grassroots level, particularly in areas lacking established dental clinics. ​

State governments throughout the nation maintain mobile dental clinic services within their public health outreach programs. The Health Minister of Goa launched the Mobile Dental Van service in 2021 to deliver dental health camps each month throughout remote regions of the state. The staff of the Goa mobile van includes expert dental surgeons who organize up to 10 monthly rural check-ups and basic treatment camps throughout different locations. The health care delivery initiative known as “Makkalai Thedi Maruthuvam,” launched by Tamil Nadu, includes mobile dental clinics alongside other health services to provide doorstep health care to the public [28]. Karnataka and Madhya Pradesh have implemented MDU programs for people living in tribal areas, along with unreachable districts within their territories. All mobile clinics run by the government operate free of charge for patients while maintaining connections with local primary health centers for further follow-up.

Government bodies are building mobile dental service programs by collaborating with non-profit organizations and private sector entities. The Smile Foundation exemplifies how state health departments work with NGO partners. Under a November 2022 partnership between GlaxoSmithKline Asia Pvt. Ltd. and Smile Foundation, four MDUs started providing services in the Delhi-NCR region as part of CSR activities. The MDUs began their operations after receiving support from local health officials. Tens of thousands of uninsured people receive free dental exams and treatment through mobile medical facilities that employ medical professionals and coordinators every year. Participation in the program establishes connections between the initiative and government medical centers for cases requiring hospital referrals to ensure continuous health care. The policy supporting collaborations between health services and the private sector results in regulatory approval assistance, supply, and patient referrals while utilizing the NGO's community outreach experience and private-sector financial support.

The Indian Dental Association (IDA), although a professional body rather than a government entity, works closely with policymakers to improve oral health in India. The IDA has been a strong advocate for improving rural oral healthcare and has explicitly called for equipping rural areas with dentists, MDUs, and tobacco cessation counselors [29]​. Through its advocacy, the IDA influences national health policy to recognize oral health needs. It often partners in government programs like school dental health initiatives and supports camps with its member dentists. This stance of the IDA reinforces to government stakeholders that mobile dental clinics are essential and should be supported through policy measures and funding. This has likely contributed to oral health being included in national health dialogues and the continuation of mobile outreach as a recommended approach.

Mobile dental clinics are increasingly being integrated into larger health missions. The National Health Mission (NHM) provides a framework for mobile medical units (MMUs) that travel to underserved areas with doctors, nurses, and sometimes dentists [30]. In some regions, the MMUs include a dental chair or have a dentist on the team to address basic dental complaints during their visits. While not a dedicated dental van, this integration ensures that dental care is not ignored in primary healthcare outreach. Additionally, India’s school health programs under the Ayushman Bharat initiative encourage health check-up drives in schools, and MDUs are invited to participate in oral health screenings.

Despite these efforts, challenges remain. A nationwide fleet of government-operated mobile dental clinics has yet to be created, and as a result, coverage can be patchy. Much of the mobile dental care is still driven by dental colleges and NGOs, with the government playing a facilitating or supportive role. However, policy trends are positive. Oral health has gained priority in public health planning, and mobile clinics are viewed as a key strategy to achieve “oral health for all.” Government funding for new MDUs has been reported in some union budgets for health, especially for aspirational districts (areas identified for focused development).

Future perspectives for mobile dentistry in India

Mobile dental clinics have demonstrated their value in India, but there is significant scope to enhance their impact and sustainability in the future. As the country strives to improve healthcare access and integrate technology, several future directions and innovations can be envisioned for mobile dentistry.

Integration of Teledentistry

One promising avenue is the incorporation of teledentistry into mobile clinic operations. With advancements in digital health, MDUs can be equipped with portable imaging devices and internet connectivity to consult with remote specialists. For example, a dentist in a mobile van could transmit dental X-rays or intraoral photographs to a specialist in a city for a second opinion or diagnosis. This enables more complex cases to be managed in coordination with experts, expanding the range of care deliverable in the field. Teledentistry can also facilitate continuing education and guidance for dentists working in isolated areas. The literature suggests mobile services “may be utilized to implement telemedicine and teledentistry services”, effectively linking rural patients with urban healthcare resources​ [31]. In the future, a mobile clinic could routinely schedule tele-consultation sessions. For instance, a periodic virtual visit by an oral surgeon or orthodontist could be arranged to triage patients who might need specialty care, thereby improving referral outcomes and patient compliance.

Solar-Powered and Smart Mobile Clinics

**Figure 3:**
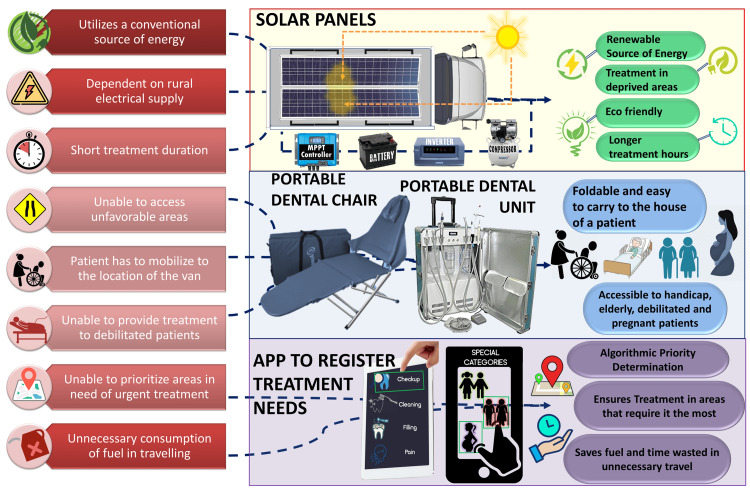
Proposed model for a solar-powered dental van with an intergrated healthcare monitoring smartphone app Image courtesy: Author Sanpreet S. Sachdev

Innovations in vehicle design and equipment can make future mobile dental clinics self-sufficient and eco-friendly. Solar panels installed on vans could power dental equipment, reducing reliance on fuel generators and ensuring a consistent power supply even in areas without electricity. Energy-efficient dental devices and better battery storage can enhance performance [32]. Additionally, future mobile clinics may be 'smart', i.e., equipped with electronic health record systems that store patient data securely and can update when the internet is available. This would help in maintaining continuity of care, as patient records from a mobile visit can be accessed at the referral hospital and vice versa. Implementing digital record-keeping also aids in data collection to evaluate program outcomes. Some pilot projects globally have tried such solar-powered mobile clinics, and adapting these for Indian conditions (sunny climate, remote villages) could be highly beneficial. The components of a ‘smart’, solar-powered MDU are depicted in Figure 3.

Expansion Through Public Health Infrastructure

Looking ahead, a systematic expansion of mobile dental clinics in India’s public health infrastructure could greatly increase coverage. This might involve dedicating a fleet of MDUs at the district level under the NHM. For instance, each district (especially those with difficult hilly or remote terrain) could have a couple of MDUs that are managed by the district hospital’s dental department and schedule visits to peripheral areas. By categorizing regions into high, medium, and low priority based on access to dental facilities, resources can be allocated more efficiently. High-need areas would get more frequent mobile clinic visits. The government could also consider an MDU program analogous to the existing MMU program, complete with monitoring and targets. This would institutionalize mobile dentistry rather than it being ad hoc. As suggested by Ganavadiya et al., pilot testing such models in select districts and then scaling up if effective would be a prudent approach​ [9].

S*trengthening Public-Private Partnerships*

Future mobile dentistry may see increased collaboration between government, academia, and private entities (corporations or non-profits). The success of partnerships like the Smile Foundation’s mobile dental vans indicates that pooling resources and expertise yields good results [33]. Corporate social responsibility funds from dental product companies or other industries could be channeled into sponsoring mobile clinics, while the public sector can provide manpower or operational support. Dental colleges will continue to be key players. Their vans and their graduates can serve the community in exchange for government incentives or as part of mandatory rural service. A public-private partnership framework could help overcome financial barriers by sharing costs, as well as improve service quality by bringing in professional management practices. In the future, we might see MDUs sponsored by companies running under the supervision of local health authorities, ensuring a win-win for community health and corporate citizenship.

Focus on Preventive and Continuous Care

The future of mobile dentistry should place even greater emphasis on prevention and continuity. Mobile clinics could evolve into 'Mobile Oral Health Units' that not only treat disease but also implement community-wide preventive programs. This could include periodic school fluoride rinse programs, community fluoride varnish drives, and distribution of oral hygiene kits during visits. Also, scheduling each community for a series of visits (e.g., a cluster of villages visited in a three-month rotation cycle) can ensure follow-up. By tracking communities over time, mobile dental teams can monitor outcomes like reduction in new cavities or improved oral hygiene, thus transitioning from just acute care to sustained oral health improvement. Some programs are already moving toward this model by educating local school teachers or community health workers to assist in follow-ups between van visits (for example, checking if referred patients went to the hospital, or reinforcing oral health messages). Expanding such integrative efforts will magnify the long-term impact of mobile clinics.

Research and Evaluation

As mobile dental services grow, there will be a need for research to evaluate their effectiveness, cost-benefit, and impact on oral health indices. Future perspectives include conducting systematic studies and perhaps establishing a centralized database for mobile clinic outcomes. Researchers have pointed out the immediate need for well-conducted studies and reviews on the effectiveness of mobile and portable dental services in developing countries like India. By gathering evidence on improvements in disease levels or patient satisfaction, policymakers can further justify investments in mobile dentistry. Additionally, data collection can help refine strategies, such as identifying which services are most utilized or which areas show the greatest improvement to guide the allocation of resources. In the coming years, one might expect academic publications from India analyzing, say, a decade of mobile clinic data to demonstrate reductions in untreated decay or extractions in target populations, strengthening the case for mobile care.

Enhanced Training and Manpower Development

The future will also require training more dentists and auxiliary personnel to excel in community and mobile dentistry. Introducing specific modules in dental curricula about managing portable equipment, field improvisation, and community engagement will prepare new dentists better. There could also be specialized training programs or fellowships in public health dentistry that focus on mobile clinic management. Furthermore, empowering mid-level dental providers (such as dental hygienists and dental assistants) to take on expanded roles in MDUs can help address workforce shortages. For instance, hygienists could independently handle preventive camps (education, cleanings, screenings) in between dentist visits. The involvement of professional organizations such as the IDA and DCI will be crucial in this aspect. As noted, they should have a strong motive to translate mobile dental service models into reality on a larger scale.

## Conclusions

Mobile dental clinics in India have emerged as a promising strategy to bridge the oral healthcare divide, particularly in rural and underserved regions. This narrative review highlights that MDUs offer multiple operational advantages, including increased geographic reach, flexibility in deployment, integration with educational and public health programs, and the ability to provide a range of preventive and basic curative services. These clinics help overcome barriers such as transportation challenges, inadequate infrastructure, and financial constraints, making them a cost-effective alternative to static dental facilities. Despite these strengths, challenges remain, such as inconsistent funding, state-wise variability in implementation under programs like the NOHP, logistical issues, and limited scope for advanced procedures.

While several studies and program reports affirm the utility and community impact of mobile dental services, there remains a lack of high-quality, large-scale data evaluating long-term outcomes, cost-benefit ratios, and patient satisfaction. Moreover, uniform guidelines and standardization of services across different states are still lacking. These gaps highlight the need for further research and policy evaluation. Globally, mobile dental units have been successfully adopted in countries such as the United States, Brazil, and South Africa to improve outreach, especially among school children, the elderly, and vulnerable populations. Insights from these international models can inform the strategic evolution of India’s mobile dental landscape.

In conclusion, the horizon for mobile dentistry in India is indeed bright, provided that efforts are grounded in data, supported by policy, and tailored to community needs. With thoughtful integration into the health system and leveraging of emerging technologies, mobile dental clinics can transition from a peripheral service to a central pillar of oral healthcare, bringing “health on wheels” to every corner of India and ensuring healthier smiles for generations to come.
